# Different Patterns of Kidney Fibrosis Are Indicative of Injury to Distinct Renal Compartments

**DOI:** 10.3390/cells10082014

**Published:** 2021-08-06

**Authors:** Désirée Tampe, Laura Schridde, Peter Korsten, Philipp Ströbel, Michael Zeisberg, Samy Hakroush, Björn Tampe

**Affiliations:** 1 Department of Nephrology and Rheumatology, University Medical Center Göttingen, 37075 Göttingen, Germany; desiree.tampe@med.uni-goettingen.de (D.T.); peter.korsten@med.uni-goettingen.de (P.K.); michael.zeisberg@med.uni-goettingen.de (M.Z.); 2 Institute of Pathology, University Medical Center Göttingen, 37075 Göttingen, Germany; laura.schridde@med.uni-goettingen.de (L.S.); philipp.stroebel@med.uni-goettingen.de (P.S.)

**Keywords:** fibrosis pattern, kidney fibrosis, kidney injury, tubular atrophy, inflammation, systemic vasculitis

## Abstract

Kidney fibrosis is a common manifestation and hallmark of a wide variety of chronic kidney disease (CKD) that appears in different morphological patterns, suggesting distinct pathogenic causes. Broad macroscopically visible scars are the sequelae of severe focal injury and complete parenchymal destruction, reflecting a wound healing response as a consequence of infarction. In the kidney, chronic glomerular injury leads to atrophy of the corresponding tubule, degeneration of this specific nephron, and finally interstitial fibrosis/tubular atrophy (IF/TA). Compared to this glomerulus-induced focal replacement scar, diffuse fibrosis independent of tubular atrophy appears to be a different pathogenic process. Kidney fibrosis appears to develop in a compartment-specific manner, but whether focal and diffuse fibrosis has distinct characteristics associated with other glomerular or tubulointerstitial lesions remains elusive. In the present study, we aimed to analyze renal fibrotic patterns related to renal lesions, which directly contribute to renal fibrogenesis, to unravel fibrotic patterns and manifestations upon damage to distinct renal compartments. Patterns of kidney fibrosis were analyzed in experimental models of CKD and various renal pathologies in correlation with histopathological and ultrastructural findings. After the induction of isolated crescentic glomerulonephritis (GN) in nephrotoxic serum-nephritis (NTN), chronic glomerular damage resulted in predominantly focal fibrosis adjacent to atrophic tubules. By contrast, using unilateral ureteral obstruction (UUO) as a model of primary injury to the tubulointerstitial compartment revealed diffuse fibrosis as the predominant pattern of chronic lesions. Finally, folic acid-induced nephropathy (FAN) as a model of primary tubular injury with consecutive tubular atrophy independent of chronic glomerular damage equally induced predominant focal IF/TA. By analyzing several renal pathologies, our data also suggest that focal and diffuse fibrosis appear to contribute as chronic lesions in the majority of human renal disease, mainly being present in antineutrophil cytoplasmic antibody (ANCA)-associated GN, lupus nephritis, and IgA nephropathy (IgAN). Focal IF/TA correlated with glomerular damage and irreversible injury to nephrons, whereas diffuse fibrosis in ANCA GN was associated explicitly with interstitial inflammation independent of glomerular damage and nephron loss. Ultrastructural analysis of focal IF/TA versus diffuse fibrosis revealed distinct matrix compositions, further supported by different collagen signatures in transcriptome datasets. With regard to long-term renal outcome, only the extent of focal IF/TA correlated with the development of end-stage kidney disease (ESKD) in ANCA GN. In contrast, diffuse kidney fibrosis did not associate with the long-term renal outcome. In conclusion, we here provide evidence that a focal pattern of kidney fibrosis seems to be associated with nephron loss and replacement scarring. In contrast, a diffuse pattern of kidney fibrosis appears to result from primary interstitial inflammation and injury.

## 1. Introduction

Kidney fibrosis is a common manifestation and the hallmark of a wide variety of chronic kidney diseases (CKD) leading to end-stage kidney disease (ESKD), regardless of the underlying etiology [1]. Generally, kidney fibrosis (or tubulointerstitial fibrosis) represents the histomorphology of extracellular matrix (ECM) deposition in all stages of CKD. Kidney fibrosis can appear in different morphological patterns, suggesting other pathogenic causes [2]. Broad macroscopically visible scars are the sequelae of severe focal injury and complete parenchymal destruction, reflecting a wound healing response as a consequence of infarction [3]. In the kidney, chronic glomerular injury leads to atrophy of the corresponding tubule, degeneration of this specific nephron, and finally interstitial fibrosis/tubular atrophy (IF/TA) [4,5]. Compared to this glomerulus-induced focal replacement scar, fibrosis independent of tubular atrophy (in the following, referred to as diffuse fibrosis) appears to be a different pathogenic process [6,7]. Tubular atrophy is defined as a loss of specialized transport and metabolic capacity and is typically characterized by small tubules, epithelial cells with pale cytoplasm, or dilated very thin tubules.

In contrast to our understanding of fibrosis as scar tissue representing an incomplete renal repair process, diffuse fibrosis is considered as an active contributor of CKD progression, which is essentially based on the observation that the decline of renal function correlates more closely with tubulointerstitial fibrosis rather than with glomerular damage [8,9,10,11]. Based on the concepts above, kidney fibrosis is discussed either as a mechanism of incomplete kidney repair or an active contributor to CKD progression [12,13]. Kidney fibrosis appears to develop in a compartment-specific manner, but whether focal IF/TA or diffuse fibrosis have distinct characteristics associated with other glomerular or tubulointerstitial lesions remains elusive. In the present study, we aimed to analyze renal fibrotic patterns related to the aforementioned renal lesions (e.g., glomerular, tubular, and interstitial lesions), which directly contribute to renal fibrogenesis.

## 2. Materials and Methods

### 2.1. Animals

All experimental animal studies were performed with the approval of the Institutional Animal Care and Use Committee of the Beth Israel Deaconess Medical Center (BIDMC) and the University Medical Center Göttingen in compliance with the ARRIVE guidelines [14]. Experimental protocols are detailed below. A sample size of *n* = 5 mice in each group was not formally powered or prespecified.

### 2.2. Nephrotoxic Serum-Nephritis (NTN)

Each mouse was initially pre-immunized with 200 μg sheep IgG (Capralogics, Gilbertville, IA, USA) in 200 μL complete Freund’s adjuvant (Sigma, St. Louis, MO, USA) intravenously injected with 40 μL nephrotoxic serum at days 5, 6 and 7 after pre-immunization. Experiments ended 63 days following immunization [15,16].

### 2.3. Unilateral Ureteral Obstruction (UUO)

Eight to twelve weeks old C57BL/6 mice were anesthetized with isoflurane inhalation, and analgesia was performed by subcutaneous buprenorphine injection. The ureter was separated from the surrounding tissues, and two ligatures were placed about 5 mm apart in the upper two-thirds of the ureter of the left kidney to obtain reliable obstruction. Experiments ended ten days after ureter ligation as described previously [17].

### 2.4. Folic Acid-Induced Nephropathy (FAN)

Kidney injury was induced with a single intraperitoneal injection of folic acid (250 mg/kg body weight in PBS) in CD1 mice. Experiments ended 96 days after injection.

### 2.5. Study Population

A total number of 112 cases of various renal pathologies including acute interstitial nephritis (AIN), antineutrophil cytoplasmic antibody-associated associated glomerulonephritis (ANCA GN), membranous GN, lupus nephritis, hypertensive nephropathy, IgA nephropathy (IgAN), focal-segmental glomerulosclerosis (FSGS), and diabetic kidney disease (DKD) were included. While no formal approval was required for the use of routine clinical data, a favorable opinion was granted by the ethics committee of the University Medical Center Göttingen (no. 22/2/14 and 28/9/17). Furthermore, all patients consented to data collection as part of their regular medical care.

### 2.6. Definitions

The estimated glomerular filtration rate (GFR) was calculated using the Chronic Kidney Disease Epidemiology Collaboration (CKD-EPI) equation [18]. For cases of ANCA GN, the Birmingham Vasculitis Activity Score (BVAS) version 3 was calculated as described previously [19]. The BVAS is assessed on a scale of 0 to 63, with a 0 indicating the absence of disease activity and higher scores indicating active disease.

### 2.7. Masson’s Trichrome Stain

Formalin-fixed, paraffin-embedded kidneys were sectioned at 3 µm, and staining was performed at the BIDMC Histopathology Core and the University Medical Center Göttingen. 

### 2.8. Renal Histopathology

Renal pathologists (L.S., S.H., and P.S.) evaluated all biopsies and were blinded to clinical data collection and analysis. Analogously to the Banff scoring system, the percentage of cortical areas affected by total kidney fibrosis and focal interstitial fibrosis in the area of tubular atrophy (IF/TA) were assessed within the entire specimen. Diffuse fibrosis not related to tubular atrophy was calculated by subtraction [20]. In addition, each glomerulus was scored separately for the presence of necrosis, crescents, and global sclerosis. Consequently, the percentage of glomeruli with any of these features was calculated as a fraction of the total number of glomeruli in each renal biopsy. Based on these scorings, histopathological subgrouping according to Berden et al. (focal, crescentic, mixed, or sclerotic class) and ARRS according to Brix et al. (low, medium, or high risk) were performed [21,22].

### 2.9. Remission Induction Therapy

Glucocorticoids (GCs) were administered either as intravenous pulse therapy or orally with a tapering schedule. Plasma exchange (PEX) was administered during the induction period at the discretion of treating physicians. Rituximab (RTX) was administered in four intravenous doses at 375 mg/m^2^ every week; RTX was not administered within 48 h before PEX treatment. Cyclophosphamide (CYC) was administered in three intravenous doses up to 15 mg/kg every two weeks and every three weeks after that, adjusted for age and renal function. Combination therapy was administered in four intravenous doses at 375 mg/m^2^ RTX every week and two intravenous doses at 15 mg/kg CYC every two weeks. At the discretion of treating physicians, remission induction therapy depended on previous regimens and individual patient factors. RTX was preferred in younger patients, with toxicity being the main reason for this choice [23]. Prophylaxis to prevent *Pneumocystis jiroveci* infection was given according to local practice.

### 2.10. Analyses of Publicly Available Array Datasets

Publicly available datasets were analyzed according to general recommendations [24]. Human transcriptome array data are shown as log_2_ median centered intensities extracted from the Nephroseq database, including the European Renal cDNA Biobank (ERCB) from 170 CKD patients and 31 healthy living donors (accession number GSE69438) [25]. 

### 2.11. Statistical Methods

Variables were tested for normal distribution using the Shapiro–Wilk test. Non-normally distributed continuous variables are expressed as the median and interquartile range (IQR), categorical variables are presented as frequency and percentage. Statistical comparisons were not formally powered or prespecified. For between groups comparison, the Kruskal–Wallis test was used. For group comparisons, the Mann–Whitney U-test was used to determine differences in medians. Non-parametric between-group comparisons were performed with Pearson’s chi-square test. Correlations were analyzed using Spearman’s rank correlation coefficient (Spearman’s ρ), and data analyses were performed with GraphPad Prism (version 8.4.3 for macOS, GraphPad Software, San Diego, CA, USA).

## 3. Results

### 3.1. Injury to Distinct Renal Compartments Results in Different Patterns of Kidney Fibrosis

**Figure 1:**
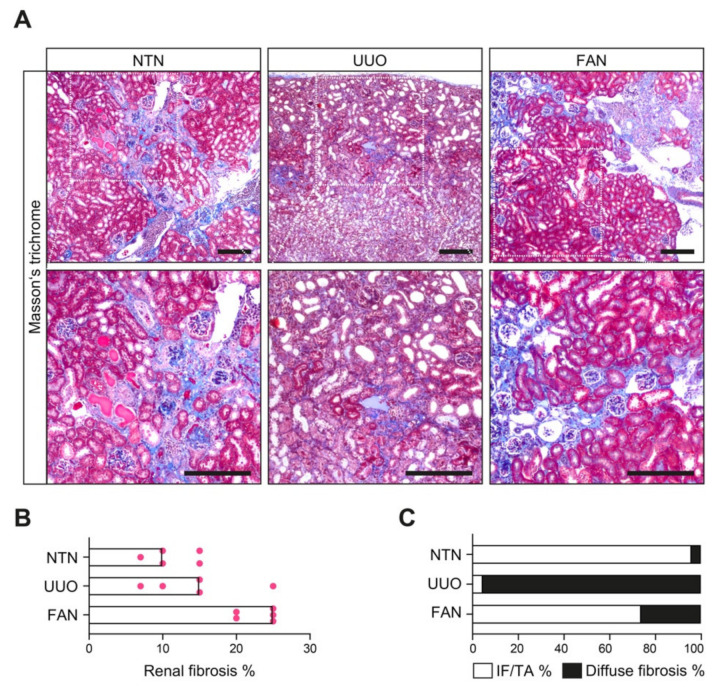
Distribution of focal IF/TA and diffuse fibrosis in experimental kidney diseases with different types of injury. (**A**) Representative photomicrograph of Masson’s trichrome-stained kidney section in mouse models of experimental kidney diseases (scale bars: 200 μm). (**B**) The scatter dot plots represent medians with individual data points summarizing the extent of total kidney fibrosis in indicated experimental models (*n* = 5 mice in each group). (**C**) The bar graph represents the fraction of focal IF/TA and diffuse fibrosis relative to total fibrosis in the indicated experimental models (*n* = 5 mice in each group). Abbreviations: FAN—folic acid-induced nephropathy; IF/TA—interstitial fibrosis/tubular atrophy; NTN—nephrotoxic serum-nephritis; UUO—unilateral ureteral obstruction.

To clarify potential underlying disease mechanisms in the development of focal IF/TA and diffuse fibrosis as a result of glomerular or tubular injury, we first challenged mice with experimental models of primary glomerular injury leading to glomerular sclerosis induced by nephrotoxic serum-nephritis (NTN), diffuse tubulointerstitial injury due to postrenal failure with renal hemodynamic and metabolic changes caused by unilateral ureteral obstruction (UUO), and a specific model of tubular injury leading to tubular atrophy by folic acid-induced nephropathy (FAN, Figure 1A) [26,27,28]. The kidneys in these experimental models of kidney injury were assessed by Masson’s trichrome staining revealing that chronic glomerular damage in NTN resulted in predominantly focal IF/TA adjacent to atrophic tubules (Figure 1B,C and Table 1). By contrast, using UUO as a model of primary injury to the tubulointerstitial compartment with associated inflammatory lesions revealed diffuse fibrosis as the leading pattern of chronic lesions (Figure 1B,C and Table 1). Interestingly, FAN as a model of primary tubular injury with consecutive tubular atrophy independent of chronic glomerular damage equally induced a predominant focal IF/TA (Figure 1B,C and Table 1), thereby underscoring a role of tubular injury and tubular atrophy for the development of this focal fibrosis pattern. 

### 3.2. Distribution of Focal IF/TA and Diffuse Fibrosis in Human Pathologies

**Figure 2:**
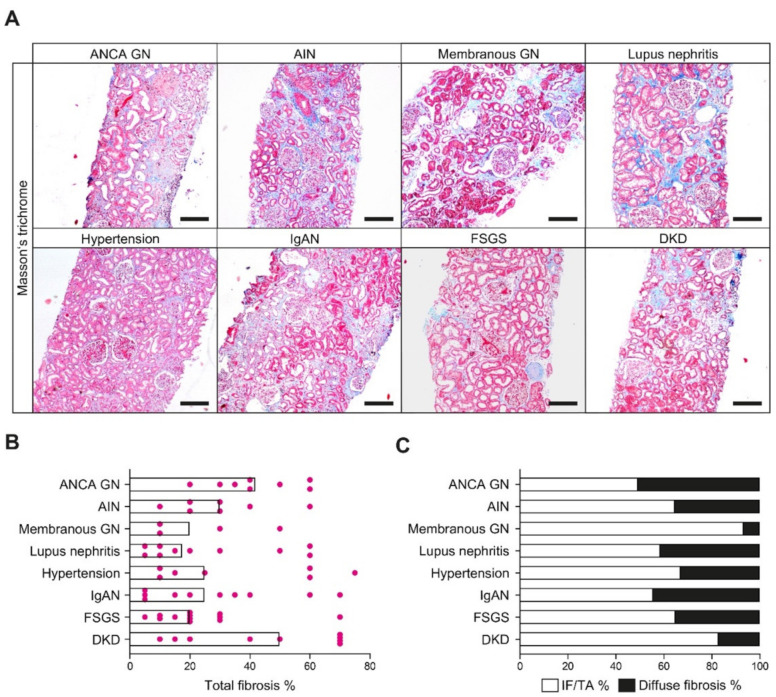
Distribution of focal IF/TA and diffuse fibrosis in various renal pathologies. (**A**) Representative photomicrograph of Masson’s trichrome-stained kidney section in various renal pathologies (scale bars: 200 μm). (**B**) The scatter dot plots represent the medians with individual data points summarizing the extent of total kidney fibrosis in indicated renal pathologies. (**C**) The bar graph summarizes the fraction of focal IF/TA and diffuse fibrosis relative to total fibrosis in the indicated renal pathologies. Abbreviations: AIN—acute interstitial nephritis; ANCA—antineutrophil cytoplasmic antibody; DKD—diabetic kidney disease; FSGS—focal segmental glomerulosclerosis; GN—glomerulonephritis; IF/TA—interstitial fibrosis/tubular atrophy; IgAN—immunoglobulin A nephropathy.

To verify whether distinct patterns of kidney fibrosis are general disease progression mechanisms in response to chronic injury and observed in renal pathologies, we next analyzed the occurrence and distribution of focal IF/TA and diffuse fibrosis in various other human kidney diseases. In a total number of 67 kidney biopsies with different renal pathologies, including antineutrophil cytoplasmic antibody-associated glomerulonephritis (ANCA GN), acute interstitial nephritis (AIN), membranous GN, lupus nephritis, hypertensive nephropathy, IgA nephropathy (IgAN), focal-segmental glomerulosclerosis (FSGS), and diabetic kidney disease (DKD), focal IF/TA and diffuse fibrosis could be observed to variable degrees (Figure 2A–C and Table 2). Interestingly, diffuse fibrosis was predominantly present in cases of ANCA GN, lupus nephritis, and IgAN as kidney diseases with glomerular and interstitial injury (Figure 2C). These findings suggest that distinct patterns of kidney fibrosis are present in various renal pathologies, including ANCA GN, and that ANCA GN could serve as a model disease for studying inflammatory and degenerative injury mechanisms to different renal compartments [29].

### 3.3. Focal IF/TA and Diffuse Fibrosis Are Indicative of Injury to Distinct Renal Compartments in ANCA GN

Because we found that distinct patterns of kidney fibrosis are present in various renal pathologies, including ANCA GN as kidney disease with glomerular and inflammatory interstitial injury with an equal distribution of focal IF/TA and diffuse fibrosis, we analyzed distinct patterns of kidney fibrosis in association with clinical, laboratory and histopathological findings within a total number of 49 renal biopsies with confirmed ANCA GN (Table 3) [30,31,32,33,34,35,36].

**Figure 3:**
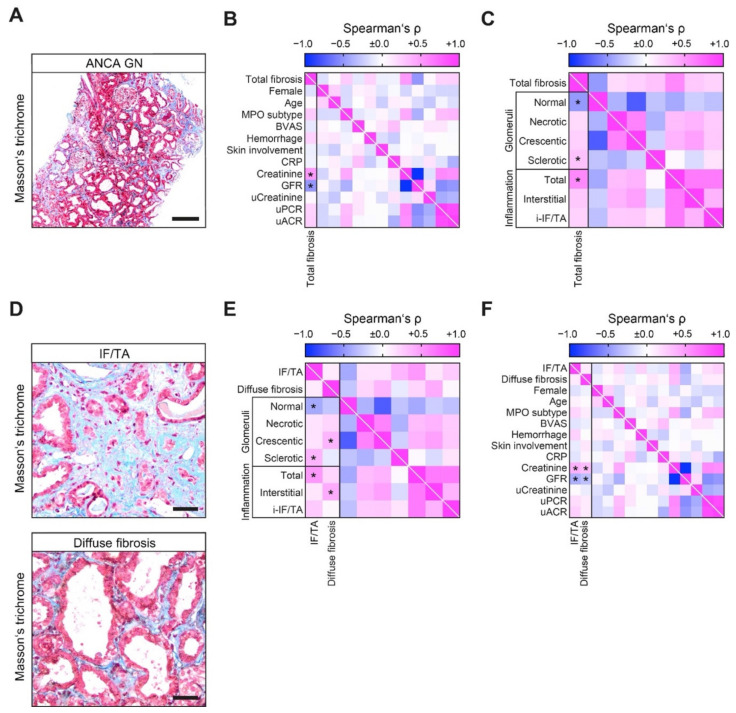
Focal IF/TA and diffuse fibrosis are indicative of injury to distinct renal compartments in ANCA GN. (**A**) Representative photomicrograph of Masson’s trichrome-stained kidney section in ANCA GN (scale bar: 200 μm). (**B**) Association between total kidney fibrosis, clinical and laboratory findings in ANCA GN are shown by heatmap reflecting mean values of Spearman’s ρ, asterisks indicate *p* < 0.05. (**C**) Association between total kidney fibrosis, glomerular and inflammatory findings in ANCA GN are shown by heatmap reflecting mean values of Spearman’s ρ, asterisks indicate *p* < 0.05. (**D**) Representative photomicrograph of Masson’s trichrome-stained kidney section in ANCA GN with focal IF/TA (upper panel) and diffuse fibrosis (lower panel) are shown (scale bars: 40 μm). (**E**) Association between focal IF/TA and diffuse fibrosis with glomerular and inflammatory findings are illustrated by heatmap reflecting mean values of Spearman’s ρ, asterisks indicate *p* < 0.05. (**F**) Association between focal IF/TA and diffuse fibrosis with clinical and laboratory findings in ANCA GN are shown by heatmap reflecting mean values of Spearman’s ρ, asterisks indicate *p* < 0.05. Abbreviations: ANCA GN—antineutrophil cytoplasmic antibody glomerulonephritis; BVAS—Birmingham Vasculitis Activity Score; CRP—c-reactive protein; GFR—glomerular filtration rate; IF/TA—interstitial fibrosis/tubular atrophy; IQR—interquartile range; MPO—myeloperoxidase; uACR—urine albumin/creatinine ration; uPCR—urine protein/creatinine ratio.

The extent of total fibrosis correlated with severe deterioration of kidney function, which was reflected by the rise of serum creatinine and loss of glomerular filtration rate (GFR, Figure 3B). By systematically scoring ANCA GN, total fibrosis correlated with a decreased fraction of normal glomeruli attributed to accelerated crescents and global glomerular sclerosis (Figure 3C). Among inflammatory lesions, total fibrosis correlated with total inflammation in ANCA GN not explicitly attributed to interstitial inflammation outside areas of IF/TA or inflammation in areas of interstitial fibrosis and tubular atrophy (i-IF/TA, Figure 3C). We next dissected kidney fibrosis into focal IF/TA and diffuse fibrosis surrounding intact tubules without prominent signs of tubular atrophy for separate analysis (Figure 3D). Interestingly, we observed no direct correlation between focal IF/TA and diffuse fibrosis (Figure 3E), implicating distinct characteristics of each lesion in ANCA GN. As we previously observed for total fibrosis, IF/TA correlated with a decreased fraction of normal glomeruli, mainly attributed to global glomerular sclerosis (Figure 3E), confirming the established mechanism that chronic glomerular injury leads to degeneration of the corresponding tubules with tubular atrophy and focal fibrotic scarring [4]. By contrast, diffuse fibrosis did not correlate with chronic glomerular damage but with crescentic glomeruli (Figure 3E), implying that diffuse fibrosis (not related to tubular atrophy) underlies yet unknown mechanisms that are independent of chronic glomerular injury and nephron loss. Systematic scoring of renal inflammation revealed that diffuse fibrosis was specifically associated with interstitial inflammation in non-fibrotic areas (Figure 3E), whereas focal IF/TA correlated with total cortical inflammation that was not specifically attributed to interstitial inflammation or i-IF/TA (Figure 3E). Of note, both lesions correlated with more severe deterioration of kidney function (Figure 3F), further supporting the observation that each lesion is an essential contributor to renal injury and outcome.

### 3.4. Analysis of Focal IF/TA versus Diffuse Fibrosis Reveals Distinct Matrix Compositions

**Figure 4:**
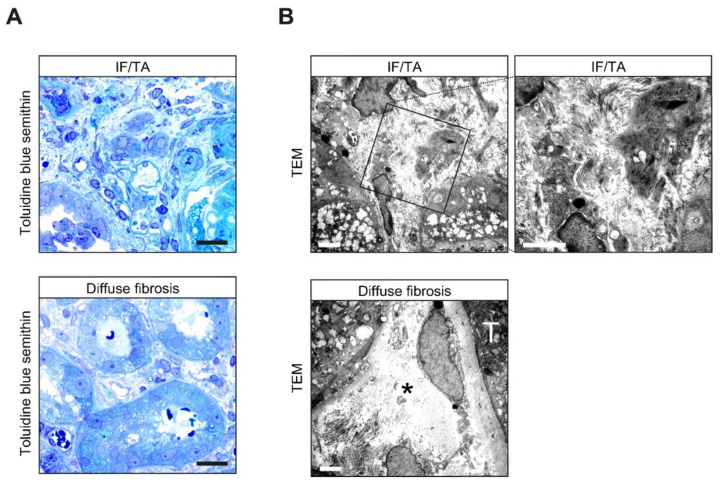
Ultrastructural analysis of focal IF/TA versus diffuse fibrosis reveals distinct matrix compositions. (**A**) Representative photomicrograph of toluidine blue-stained semithin kidney section in ANCA GN with focal IF/TA (upper panel) and diffuse fibrosis (lower panel) are shown (scale bars: 20 μm). (**B**) Interstitial composition analyzed by TEM revealed collagen bundles embedded in a densely packed stromal matrix in focal IF/TA. By contrast, ultrastructural analysis of diffuse fibrosis showed intact tubules (T) surrounded by a widened edematous interstitium (asterisk) with focal collagenous bundles revealed incomplete fibrotic tissue in ANCA GN (scale bars: 2000 nm). Abbreviations: IF/TA—interstitial fibrosis/tubular atrophy; TEM—transmission electron microscopy.

Next, we analyzed the morphological characteristics of focal IF/TA and diffuse fibrosis in ANCA GN in more detail. Semithin sections revealed that focal IF/TA surrounding atrophic tubules was more condensed than the loosely arranged fibrotic tissue surrounding preserved intact tubules (Figure 4A). Transmission electron microscopy (TEM) elucidated collagen bundles embedded in a densely packed stromal matrix (Figure 4B). By contrast, ultrastructural analysis of diffuse fibrosis showed almost intact tubules surrounded by a widened edematous interiority with focal collagenous bundles revealing an incomplete kind of fibrotic tissue (Figure 4B). These observations implicated that focal IF/TA and diffuse kidney fibrosis may differ in terms of ECM composition and organization.

### 3.5. Tubulointerstitial Transcriptome in Association with Tubular Atrophy Reveals Distinct Collagen Signatures in Kidney Fibrosis

**Figure 5:**
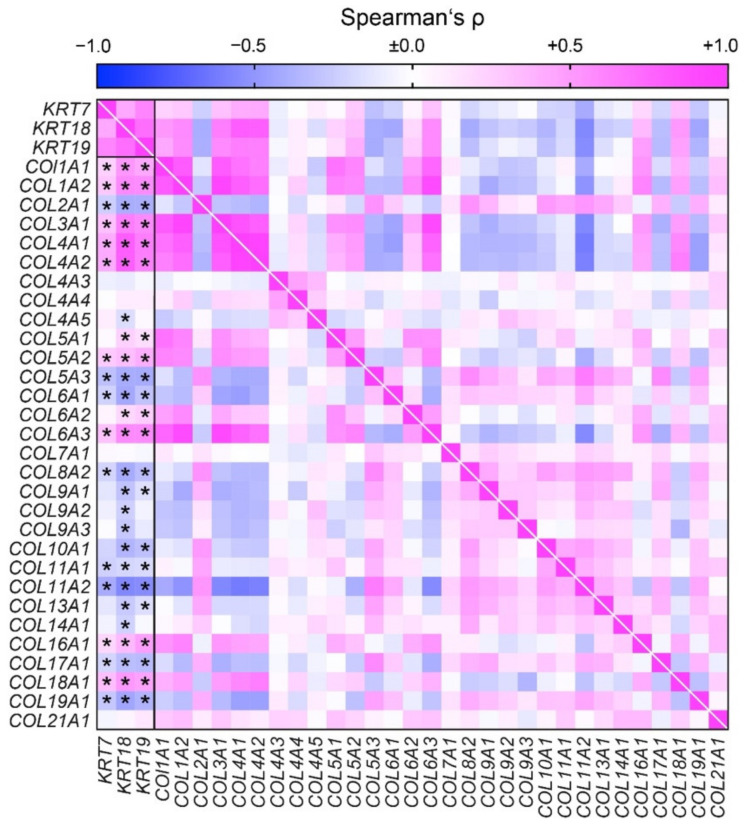
Tubulointerstitial transcriptome in association with tubular atrophy reveals distinct collagen signatures in kidney fibrosis associated with tubular injury markers. Association between mRNA expression of tubular atrophy markers (*KRT7*, *KRT18*, and *KRT19*) and various collagens are shown by heatmap reflecting mean values of Spearman’s ρ of log_2_ median centered intensities extracted from Nephroseq database (accession number GSE69438), asterisks (*) indicate *p* < 0.05.

We next analyzed the ECM composition reflected by collagen signatures in transcriptome datasets from microdissected tubulointerstitial compartments in 170 CKD patients and 31 healthy living donors (Nephroseq database) for an association with established markers of tubular atrophy, including cytokeratin-7 (encoded by *KRT7*), -18 (*KRT18*), and -19 (*KRT19*) [25,37]. In tubulointerstitial compartments with high expression of tubular injury markers, collagens *COL1A1*, *COL1A2*, *COL3A1*, *COL4A1*, *COL4A2*, *COL5A2*, *COL6A3*, *COL16A1,* and *COL18A1* were predominantly expressed (Figure 5). By contrast, an inverse correlation was observed for *COL2A1*, *COL5A3*, *COL6A1*, *COL8A2*, *COL11A1*, *COL11A2*, *COL17A1,* and *COL19A1* (Figure 5). These results implicate that kidney fibrosis may have distinct collagen signatures in markers of tubular injury.

### 3.6. Focal IF/TA Is Associated with Worse Long-Term Outcome in ANCA GN

To gain insights into whether distinct patterns of kidney fibrosis are associated with long-term renal outcomes, we next compared total, focal IF/TA, and diffuse renal fibrosis in ANCA GN with the development of ESKD. Total kidney fibrosis in ANCA GN correlated with worse long-term renal outcomes (Table 4). Interestingly, only the extent of focal kidney fibrosis correlated with renal outcomes (Table 4). In contrast, diffuse kidney fibrosis did not associate with long-term renal outcomes (Table 4), implying that distinct fibrosis patterns may also impact the disease progression and potential treatment response.

## 4. Discussion

To date, the development of kidney fibrosis can be separated into two concepts: on the one hand, the literature provides solid evidence that kidney fibrosis is the simple consequence of irreversible damage to the nephron, either due to chronic glomerular or tubular injury, both resulting in tubular atrophy and focal interstitial fibrosis surrounding damaged nephrons [12,13]. Thus, focal IF/TA associated with tubular atrophy contributes to kidney repair by serving as scar tissue, thereby replacing nephrons that are already lost [12]. On the other hand, kidney fibrosis may be seen as an active, progressive, and damaging remodeling process of the renal interstitium. In this process, fibroblasts directly contribute to epithelial injury, suggesting a genuine interstitial mechanism of CKD progression [13].

To our knowledge, this is the first study that systematically dissects kidney fibrosis into two distinct and independent manifestations with focal or diffuse histomorphological patterns, thereby elucidating two different pathogenic pathways of these lesions. To provide new insights into the field of renal fibrogenesis, the development and fate of kidney fibrosis, we examined three different mouse models with known entities of kidney fibrosis to unravel fibrotic patterns and manifestations upon damage to distinct renal compartments. Induction of an isolated crescentic GN in NTN, and chronic glomerular damage, resulted in predominantly focal fibrosis adjacent to atrophic tubules [26]. These observations support the concept of irreversible nephron damage that results in focal IF/TA surrounding damaged nephrons [12,13]. By contrast, using UUO as a model of primary injury to the tubulointerstitial compartment with associated inflammatory lesions revealed diffuse fibrosis as the leading pattern of chronic lesions [27]. Kidney fibrosis in UUO mice was characterized by delicate collagen fibers surrounding almost intact tubules.

Interestingly, the interstitium of UUO mice showed not only diffuse fibrosis but also abundant inflammatory cells. Since inflammation is a response to injury, it is important to discuss whether interstitial inflammation results from direct damage to the interstitial compartment independent of the neighboring nephron. Consequently, diffuse fibrosis results from isolated interstitial nephritis without a primary injury to the renal epithelial parenchyma, which can occur in later stages of disease progression as tubulointerstitial nephritis, and probably leads to atrophic nephrons. Urine congestion within the tubular epithelial system is the primary culprit within the UUO model, leading to diffuse fibrosis, renal hemodynamic, and metabolic changes [27]. Since every congestion of the parenchyma leads to edema of the surrounding interstitium, diffuse edematous injury of the renal interstitium could explain the extent of the inflammation and diffuse fibrosis in UUO mice. Finally, FAN as a model of primary tubular injury with consecutive tubular atrophy independent of chronic glomerular damage equally induced a predominant focal IF/TA, thereby underscoring a role of tubular injury and tubular atrophy for the development of focal IF/TA [28]. In this model, multiple neighboring nephrons appeared to be injured by direct tubular toxicity, leading to extended tubular atrophy without glomerular damage.

Following the analysis of several renal pathologies, our data also suggest that focal IF/TA and diffuse fibrosis appear to contribute as chronic lesions in the majority of human renal disease. Focal IF/TA and diffuse fibrosis are mainly present in ANCA GN and underscore the postulated mechanism of direct interstitial injury as the cause of diffuse fibrosis. In ANCA GN as kidney disease with glomerular injury (crescent formation, glomerular sclerosis, and nephron loss) and interstitial injury (due to inflammation), we here provide evidence that tubulointerstitial fibrosis is either associated with nephron damage (dependent or independent of glomerular scarring) or primary interstitial injury (leading to a diffuse fibrotic interstitial remodeling) [22,38,39,40,41]. Furthermore, our data show that focal IF/TA in ANCA GN correlated with global glomerular sclerosis, resulting in glomerular sclerosis and nephron loss [26]. Here, we could also reveal that focal renal scarring represents the central pattern of kidney fibrosis, confirming a great abundance of literature describing that chronic glomerular damage leads to degeneration of the corresponding tubule with tubular atrophy, loss of this specific nephron, and incomplete kidney repair by a focal replacement scar [12]. Thus, our data confirm that focal IF/TA is associated with irreversible injury to nephrons, either dependent or independent of glomerular injury. The focal IF/TA patterns in human biopsies appear to be of a similar origin, reflecting kidney repair. By contrast, diffuse fibrosis in ANCA GN was specifically associated with interstitial inflammation. Semithin sections revealed fibrotic tissue with loosely arranged collagen fibers surrounding almost completely intact tubules lacking signs of true atrophy. Of note, TEM analysis showed very sparsely arranged collagen bundles within a very loosely organized interstitium, suggesting edematous interstitial changes. Because ANCA GN is a vasculitis affecting small vessels, interstitial inflammation and diffuse fibrosis could be explained due to interstitial vasculitis with an accompanied capillary leak; thereby, edema and interstitial inflammation aggravate diffuse interstitial fibrosis [42].

Transcriptome datasets from microdissected tubulointerstitial compartments revealed distinct collagen signatures associated with focal IF/TA associated with tubular atrophy and diffuse fibrosis not related to tubular atrophy, implying that focal IF/TA and diffuse kidney fibrosis may differ in ECM composition. Collagen is a major abundant fibrous protein in the extracellular matrix. Collagens constitute the primary structural element of the ECM and provide tensile strength, regulate cell adhesion, support chemotaxis and migration, and direct tissue development [43]. To date, 28 types of collagen have been described. The main types of collagen found in connective tissues are types I, II, III, V, and XI. In microdissected tubulointerstitial compartments, focal fibrosis associated with markers of tubular atrophy predominantly contained collagen types I, III, IV, and V. By contrast, we identified an inverse correlation with collagen types II and XI, implying that focal IF/TA and diffuse kidney fibrosis may differ in distinct collagen signatures.

Finally, we here show that total kidney fibrosis in ANCA GN correlated with worse long-term renal outcomes, as described previously [22]. Interestingly, only the extent of focal kidney fibrosis correlated with renal outcome. In contrast, diffuse kidney fibrosis did not associate with the long-term renal outcome. These observations implicate that distinct fibrosis patterns may also impact disease progression and potentially treatment response.

Taken together, we here provide evidence that the majority of kidney fibrosis appears to be associated with nephron loss and replacement scarring, representing incomplete tissue repair. By contrast, diffuse fibrosis seems to be the result of primary interstitial inflammation and injury without damage to the epithelial compartment of the kidney. Interestingly, both patterns of fibrosis correlated with more severe deterioration of kidney function, implying that each interstitial fibrotic lesion is an essential contributor to the outcome. At the same time, the concept of a loss of functional parenchyma leading to focal replacement fibrosis and diffuse fibrosis underlying alternative mechanisms has not yet been systematically described in the kidney. Such mechanisms have, however, been reported in cardiac fibrosis. Replacement fibrosis (scar fibrosis) is focal and occurs after cardiomyocyte necrosis, for example, after myocardial infarction, and is considered irreversible to prevent cardiac muscle rupture after infarction [44]. On the other hand, diffuse fibrosis has been associated with the diffuse spread of extracellular collagen without cardiomyocyte necrosis and is believed to be reversible in principle [45,46,47,48].

The main limitations of our study are the limited number of kidney biopsies in some renal pathologies, its retrospective design, and the limited transferability of experimental models of kidney injury into humans. Nevertheless, our main finding that focal IF/TA and diffuse fibrosis are independent patterns of kidney fibrosis in ANCA GN (and other chronic kidney diseases) implies that each lesion has distinct characteristics and, probably, mechanisms in ANCA GN. Furthermore, these observations further expand our current knowledge of the interplay between inflammation, renal injury, and fibrosis, contributing to a more precise understanding of inflammatory responses and potential novel therapeutical strategies to modulate distinct manifestations of kidney fibrosis. 

## 5. Conclusions

In conclusion, we here provide evidence that the focal pattern of kidney fibrosis seems to be associated with nephron loss and replacement scarring. In contrast, the diffuse pattern of kidney fibrosis appears to result from primary interstitial inflammation and injury.

## Figures and Tables

**Table 1:** Distribution of total fibrosis, focal IF/TA, and diffuse renal fibrosis in experimental kidney diseases with different types of injury.

Model	% Total Fibrosis	% Focal IF/TA	% Diffuse Fibrosis
NTN (*n* = 5)	10 (8.5–15)	10 (8.5–13.5)	0 (0–1.5)
UUO (*n* = 5)	15 (8.5–20)	0 (0–2)	15 (11.5–20)
FAN (*n* = 5)	25 (20–25)	15 (15–20)	5 (5–7.5)
*p* value	0.0123	<0.0001	<0.0001

Median values and IQR are shown. The Kruskal–Wallis test was used for between-group comparison. Abbreviations: FAN—folic acid-induced nephropathy; NTN—nephrotoxic serum-nephritis; UUO—unilateral ureteral obstruction.

**Table 2:** Distribution of total fibrosis, focal IF/TA, and diffuse renal fibrosis among various renal pathologies.

Renal Disease	% Total Fibrosis	% Focal IF/TA	% Diffuse Fibrosis
ANCA GN (*n* = 8)	25 (12.5–45)	15 (5–37.5)	5 (5–10)
AIN (*n* = 7)	30 (20–40)	20 (7–25)	5 (0–20)
Membranous GN (*n* = 4)	20 (10–45)	17.5 (10–40)	2.5 (0–5)
Lupus nephritis (*n* = 10)	17.5 (8.75–52.5)	7.5 (5–31.3)	5 (2.75–11.3)
Hypertension (*n* = 7)	25 (10–60)	15 (5–40)	5 (5–20)
IgAN (*n* = 10)	25 (5–45)	17.5 (0.75–36.3)	5 (2.25–12.5)
FSGS (*n* = 12)	20 (11.3–30)	14 (5–25)	5 (2–10)
DKD (*n* = 9)	50 (17.5–70)	40 (12.5–60)	10 (5–10)
*p* value	0.2355	0.3009	0.0185

Median values and IQR are shown. The Kruskal–Wallis test was used for between groups comparison. Abbreviations: AIN—acute interstitial nephritis; ANCA—antineutrophil cytoplasmic antibody; DKD—diabetic kidney disease; FSGS—focal segmental glomerulosclerosis; GN—glomerulonephritis; IF/TA—interstitial fibrosis/tubular atrophy; IgAN—immunoglobulin A nephropathy.

**Table 3:** Total patient cohort of ANCA GN.

Parameter	Value
Female sex—no. (%)	22 (44.9)
Age (IQR)—years	66 (55–74.5)
MPO subtype—no. (%)	25 (51)
BVAS (IQR)—points	18 (15–21)
Pulmonary hemorrhage—no. (%)	7 (14.3)
Skin involvement—no. (%)	7 (14.3)
CRP (IQR)—mg/L	63.6 (23.4–109)
Serum creatinine (IQR)—μmol/L	272 (123–437)
GFR (IQR)—mL/min/1.73 m^2^	17.6 (9.7–47.9)
uCreatinine (IQR)—mg/dL	72.7 (41.1–98.1)
uPCR (IQR)—mg/g creatinine	977 (573–1939)
uACR (IQR)—mg/g creatinine	458 (202–938)
Total glomeruli (IQR)—no.	17 (11–28)
Normal glomeruli (IQR)—%	45.5 (25.2–73)
Glomerular necrosis (IQR)—%	15.2 (0–44.7)
Glomerular crescents (IQR)—%	33.3 (10–55.1)
Glomerular sclerosis (IQR)—%	5.1 (0–26.5)
Focal class—no. (%)	23 (46.9)
Crescentic class—no. (%)	16 (32.7)
Sclerotic class—no. (%)	3 (6.1)
Mixed class—no. (%)	7 (14.3)
Low risk—no. (%)	18 (36.7)
Medium risk—no. (%)	23 (46.9)
High risk—no. (%)	8 (16.3)
Total kidney fibrosis (IQR)—%	25 (12.5–45)
IF/TA (IQR)—%	15 (5–37.5)
Diffuse fibrosis (IQR)—%	5 (5–10)
Total inflammation (IQR)—%	10 (5–20)
Interstitial inflammation (IQR)—%	3 (1–5)
i-IF/TA (IQR)—Banff score	2 (1–3)

Median values and IQR are shown. Abbreviations: ANCA GN—antineutrophil cytoplasmic antibody glomerulonephritis; BVAS—Birmingham Vasculitis Activity Score; CRP—c-reactive protein; GFR—glomerular filtration rate; IF/TA—interstitial fibrosis/tubular atrophy; IQR—interquartile range; MPO—myeloperoxidase; uACR—urine albumin/creatinine ration; uPCR—urine protein/creatinine ratio.

**Table 4:** The long-term renal outcome in ANCA GN.

Parameter	ESKD	No ESKD	*p*-Value
Intravenous steroid pulse—no. (%)	6 (100)	28 (65.1)	
Oral GCs—no. (%)	6 (100)	43 (100)	
PEX—no. (%)	4 (66.7)	15 (34.9)	
Sessions of PEX (IQR)—no.	5 (5–7)	5 (4.25–5)	
RTX—no. (%)	3 (50)	13 (30.2)	
CYC—no. (%)	3 (50)	21 (48.8)	
RTX/CYC—no. (%)	0 (0)	7 (16.3)	
Follow-up (IQR)—days	214 (24.75–1216)	392 (94–745)	0.5816
Total fibrosis (IQR)—%	55 (43.75–65)	20 (10–40)	0.0006
Focal IF/TA (IQR)—%	50 (23.75–65)	10 (5–30)	0.0016
Diffuse fibrosis (IQR)—%	2.5 (0–21.25)	5 (5–10)	0.5092

Median values and IQR are shown. For group comparisons, the Mann–Whitney U-test was used to determine differences between medians. In addition, non-parametric between-group comparisons were performed with Pearson’s Chi-square test. Abbreviations: ANCA GN—antineutrophil cytoplasmic antibody glomerulonephritis; CYC—cyclophosphamide; GC—glucocorticoids; IF/TA—interstitial fibrosis/tubular atrophy; PEX—plasma exchange; RTX—rituximab.

## Data Availability

De-identified data are available on reasonable request from the corresponding author.
